# A new method of ventilation inhomogeneity assessment based on a simulation study using clinical data on congenital diaphragmatic hernia cases

**DOI:** 10.1038/s41598-022-27027-8

**Published:** 2022-12-31

**Authors:** Barbara Stankiewicz, Magdalena Mierzewska-Schmidt, Krzysztof Jakub Pałko, Artur Baranowski, Marek Darowski, Maciej Kozarski

**Affiliations:** 1 Department of Modeling and Supporting of Internal Organs Functions, Nalecz Institute of Biocybernetics and Biomedical Engineering, Polish Academy of Sciences, 02-109 Warsaw, Poland; 2 Department of Paediatric Anaesthesiology and Intensive Therapy, Pediatric Teaching Clinical Hospital of Medical University of Warsaw, 02-091 Warsaw, Poland

**Keywords:** Computer modelling, Respiratory tract diseases, Biomedical engineering

## Abstract

Congenital Diaphragmatic Hernia (CDH) is a diaphragm defect associated with lung hypoplasia and ventilation inhomogeneity (VI). The affected neonates are usually born with respiratory failure and require mechanical ventilation after birth. However, significant interindividual VI differences make ventilation difficult. So far, there are no clinical methods of VI assessment that could be applied to optimize ventilation at the bedside. A new VI index is a ratio of time constants T_1_/T_2_ of gas flows in both lungs. Pressure-controlled ventilation simulations were conducted using an infant hybrid (numerical-physical) respiratory simulator connected to a ventilator. The parameters of the respiratory system model and ventilator settings were based on retrospective clinical data taken from three neonates (2, 2.6, 3.6 kg) treated in the Paediatric Teaching Clinical Hospital of the Medical University of Warsaw. We searched for relationships between respiratory system impedance (Z) and ventilation parameters: work of breathing (WOB), peak inspiratory pressure (PIP), and mean airway pressure (MAP). The study showed the increased VI described by the T_1_/T_2_ index value highly correlated with elevated Z, WOB, PIP and MAP (0.8–0.9, the Spearman correlation coefficients were significant at P < 0.001). It indicates that the T_1_/T_2_ index may help to improve the ventilation therapy of CDH neonates.

## Introduction

Congenital diaphragmatic hernia (CDH) occurs in 1 of 2500–3000 live births and has a high mortality of 22–62% in severe forms^1,2^. The vast majority of CDH newborns require immediate mechanical ventilation. The main goal of the therapy is to establish gentle, lung-protective, yet effective ventilation. As the degree of lung hypoplasia and ventilation inhomogeneity (VI) is highly variable in particular patients, ventilation therapy requires an individualized approach.

VI can be defined as non-uniform, uneven ventilation of both lungs or different lung compartments. It may result from asymmetrical airway structure causing different airway resistances and/or lung compliances, non-uniform pulmonary tissue leading to disturbances in convective and diffusive gas transport, as well as ventilation/perfusion mismatch. In minor lung hypoplasia and VI, ventilation may not present any difficulties. In the most severe cases with only one functional lung (often underdeveloped), achieving acceptable gas exchange is extremely challenging. In CDH, the compressed, ipsilateral lung is smaller than expected, with less developed respiratory structures and blood vessels^3^. Both lungs are hypoplastic and differ in resistive-compliance properties that causes VI. Too aggressive ventilation may lead to ventilatory-induced lung injury (VILI) and increased morbidity and mortality; particularly pneumothorax is an independent risk factor of mortality^4–6^. Because of that, protective lung ventilation with limited peak inspiratory pressure (PIP) up to 25 ± 2 cmH_2_O, positive end-expiratory pressure (PEEP) of 3–5 cmH_2_O, high respiratory rates (RR) of 40–60 bpm, permissive hypercapnia (pCO_2_: 50–70 mmHg) and acceptance of pre-ductal SaO_2_ targets of 85–95% reduces the risk of VILI and improves survival in this group of patients^6,7^.

Despite these general recommendations there are no accurate and objective clinical methods of assessment of lung hypoplasia and VI. Therefore, the treating physician has very limited tools to adjust ventilator settings to the individual patient’s lung pathology^8^.

In our previous pilot study^9^, we proposed a new index of VI—T_1_/T_2_ that is a ratio of time constants of gas flows in both lungs (R_1_·C_1_/R_2_·C_2_), and we put forward some ideas on how it can be used in clinical conditions. The T_1_/T_2_ index pertains to the two-compartments of the RLC (R—resistance, L—inertance, C—compliance) respiratory system model^10^, numerically implemented in the Infant Hybrid Respiratory Simulator (IHRS)^10,11^.

This study aimed to assess the application of the T_1_/T_2_ index as a tool to optimize ventilation therapy of CDH neonates under laboratory conditions and based on retrospective clinical data obtained from three babies treated in the Paediatric Intensive Care Unit (PICU) of the Department of Paediatric Anaesthesiology and Intensive Therapy, in the Paediatric Teaching Clinical Hospital of the Medical University of Warsaw. The relationships between respiratory system impedance (Z), work of breathing (WOB_vt_), peak inspiratory pressure (PIP) and mean airway pressure (MAP) obtained during conventional mechanical ventilation (CMV), and the T_1_/T_2_ index for different respiratory system parameters and ventilation settings, were examined.

The study hypotheses were as follows:

- there is a significant correlation between respiratory system impedance (Z) and VI degree (T_1_/T_2_)
- there is a significant correlation between WOB, PIP, MAP and VI degree (T_1_/T_2_)
- the correlation coefficients depend on respiratory rate (RR)
- the correlation coefficients depend on the ratio C_w_/C_L_ between chest wall compliance (C_w_) and lung compliance (C_L_).

## Study design

The simulation study using the Infant Hybrid Respiratory Simulator (IHRS) was conducted at the Nalecz Institute of Biocybernetics and Biomedical Engineering, Polish Academy of Sciences (IBBE PAS), Warsaw, Poland.

The ventilation parameters of the three enrolled neonates were collected in the Paediatric Intensive Care Unit (PICU) of the Department of Paediatric Anaesthesiology and Intensive Therapy at the Paediatric Teaching Clinical Hospital of the Medical University of Warsaw, Poland. Following the current Polish law and the Declaration of Helsinki, the study was registered by the Bioethics Committee of the Medical University of Warsaw (AKBE/141/22).

### Statement on Bioethics Committee approval and informed consent

Ethical aproval was requested from the Bioethics Committee of the Medical University of Warsaw. However, it was judged by the committee as not required as the data collection on ventilation parameters was retrospective. The committee decided the study did not fulfil the criteria of the medical experiment. As a result, also the informed consent of the parents of ventilated babies was waived by the Bioethics Committee.

### Study population

The demographic and clinical characteristics of the three enrolled patients with isolated CDH were included in Table 1.

**Table 1:** Demographic data of patients.

Patient	Sex	GA (weeks)	BW (kg)	Apgar Score (1–10)	o/e LHR (%)	Liver	Defect size (A-D)^14^/patching	ETT (mm)	Mechanical ventilation (days)	Hospital stay (days)	Discharge/follow up
P1	F	34	2	5–6	21	up	C-D/Yes	3	46	97	Alive/ No
P2	F	33 + 4 days	2.6	8–7–8	- (*)	down	B/No	3	6	21	Alive/ Yes
P3	F	38	3.6	7–8	28	up	C/Yes	3.5	12	29	Alive/ Yes

*CDH diagnosis—1 week before delivery. *GA* gestational age, *BW* body weight. *ETT* endotracheal tube size, *O/e LHR* observed to expected lung to head ratio.

Children involved in the study were assessed for eligibility criteria.

The inclusion criteria are listed below:

Newborns with isolated (one-side) congenital diaphragmatic hernia;

Patients undergoing ventilator therapy using conventional ventilation;

Patients with a planned hernia repair.

The exclusion criteria are listed below:

Patients undergoing High Frequency Ventilation (HFV);

Patients undergoing ECMO therapy.

In the PICU of the Department of Paediatric Anaesthesiology and Intensive Therapy at the Paediatric Teaching Clinical Hospital of the Medical University of Warsaw, when conventional ventilation fails children are switched to HFV, if HFV also fails we consider ECMO. The study was focused on the CMV that is the recommended first-choice therapy in CDH neonates^6,7^.

### Study outcomes

The parameters of the respiratory system and mechanical ventilation were collected by the anesthesiologist (one of the researchers), from the ventilator monitor at the bedside, in the Paediatric Intensive Care Unit (PICU).

The simulation data of the study were collected in the laboratory of IBBE PAS, where the received data sets were graphically presented and statistically analyzed. The final results were discussed by researchers from both centres.

### Outcome measures

The clinical respiratory parameters of the three enrolled neonates were assessed in the stage of patient stabilization, before hernia repair. There were the parameters usually set and measured during conventional ventilation, obtained using Fabian ventilator SW 3.4 (ACUTRONIC Med. Sys. AG, Hirzel, Switzerland).

During laboratory simulations, the same as in clinic respiratory parameters were received by using Puritan Bennet 840 Ventilator, and some additional parameters—using NICO 7300 respiratory monitor, e.g. WOB_vt_, total R and C of the respiratory system.

The assessed parameters:

- BW—body weight, in kg;
- R—total dynamic resistance of the respiratory system, in cmH_2_O s/l;
- C—total dynamic compliance of the respiratory system, in ml/cmH_2_O;
- MV—minute ventilation, in l;
- RR—respiratory rate, in bpm;
- PIP—peak inspiratory pressure, in cmH_2_O;
- MAP—mean airway pressure, in cmH_2_O;
- PEEP—positive end-expiratory pressure, in cmH_2_O;
- I:E—inspiration to expiration time ratio;
- WOB_vt_—work of breathing done by a ventilator, in J/l;
- Z—respiratory system impedance, in cmH_2_O⋅s/l;
- Spearman correlations coefficients (R_s_) for dependencies: Z vs T_1_/T_2_, WOB_vt_ vs T_1_/T_2_, PIP vs T_1_/T_2_, MAP vs T_1_/T_2_, WOB_vt_ vs Z, PIP vs Z, MAP vs Z, WOB_vt_ vs PIP, WOB_vt_ vs MAP;
- The equations of the curves for these dependencies and determination coefficients (R^2^).

### Laboratory set-up

**Figure 1:**
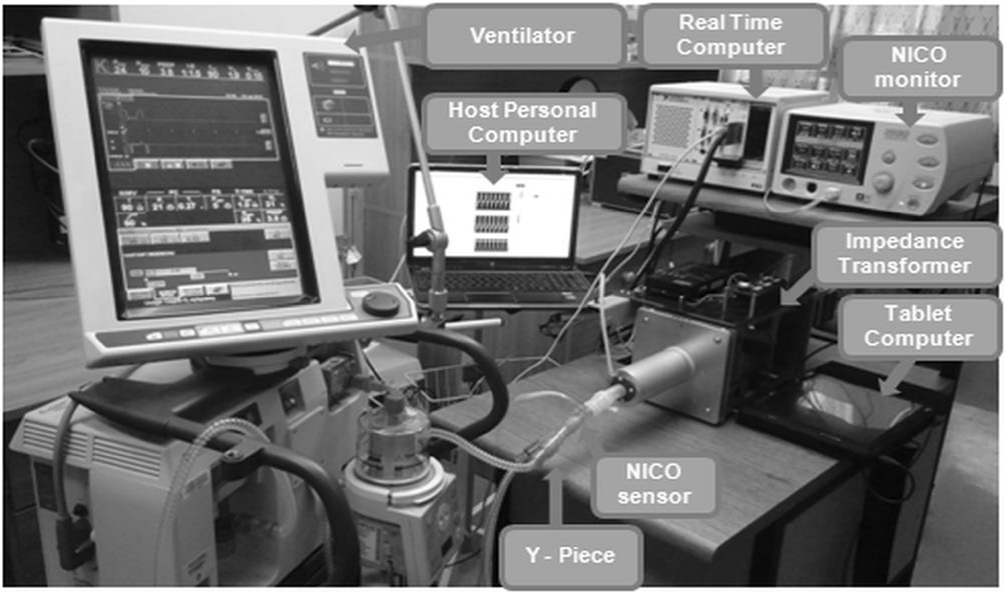
Measuring set-up. Infant Hybrid Respiratory Simulator (IHRS), ventilator and NICO monitor of respiratory parameters sent to Tablet Computer. IHRS consists of an Impedance transformer, Real-Time Computer and Host Personal Computer.

The laboratory study was carried out using Infant Hybrid Respiratory Simulator (IBBE, PAS, Warsaw, Poland), the Puritan Bennett 840 Ventilator (Medtronic, Fridley, MI, USA) and NICO 7300 respiratory monitor (Respironics Corp., Murrysville, PA, USA) (Fig. 1).

IHRS is a measurement-control platform with LabVIEW Real-Time Professional Development System 2013 (National Instruments Co., Austin, TX, USA) that coordinates the work of the numerical and physical part of the simulator and its interaction with a ventilator (Fig. 1).

**Figure 2:**
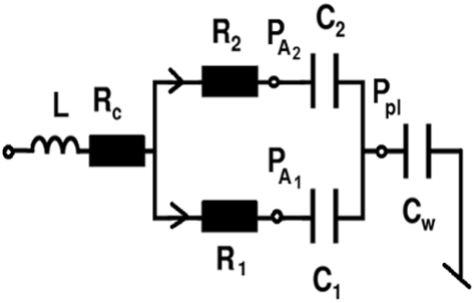
Respiratory system model. L, R_c_—central airway inertance and resistance. R_1_, R_2_, C_1_, C_2_—peripheral airway resistances and the compliance of the contralateral and ipsilateral lung. C_w_—chest wall compliance. P_A1_, P_A2_—alveolar pressure in the contralateral and ipsilateral lung. P_pl_—pleural pressure.

A numerical part of the IHRS is based on the equations set of the lung model shown in Fig. 2^10^. Using this model, it is possible to simulate different lung pathologies associated with peripheral and central airway obstruction/ restriction, the changes in C_w_ and VI^10,11^.

The physical part of IHRS is the Impedance Transformer of a cylinder-piston construction. It uses AD and DA converters that linearly transform back and forth digital signals of gas pressure and flow, obtained from lung model equations, into real physical signals of pressure and flow.

### Simulation study protocol

Based on clinical data (Table 1), the simulations of pressure-controlled ventilation (PCV)—the mode of conventional mechanical ventilation, for different T_1_/T_2_ index values (1, 1.5, 2, 3, 4, 5, 7, 10, 100) describing lung inhomogeneity, were carried out with the following ventilator settings: inspiration to expiration ratio (I:E) of 1:1.5–1:2, respiratory rate (RR) of 35, 40, 45, 50 and 55 bpm, and positive end-expiratory pressure (PEEP) equal to 4–6 cmH_2_O. Minute ventilation (MV) of 0.8 ± 0.1 l was kept constant for different combination settings, for each patient (P_1_, P_2_, P_3_). In Table 2, there are clinical data that were used for ventilator settings.

**Table 2:** Clinical data used in the simulations.

Patient	BW (kg)	R (cmH_2_O s/l)	C (ml·cmH_2_O^−1^)	Ventilator settings			
				MV (l)	PEEP (cmH_2_O)	RR (bmp)	I:E
P_1_*	2	244	0.8	0.69	4	45	1:2
P_2_	2.6	188	1.26	0.86	6	50	1:2
P_3_	3.6	205	1.14	0.9	5	45	1:2

*P_1_, P_2_, P_3_—enrolled patients, *BW* body weight, *R* total respiratory system resistance, *C* total compliance of respiratory system, *MV* minute ventilation, *PEEP* positive end-expiratory pressure, *RR* respiratory rate, *I:E* inspiratory-to-expiratory time ratio.

WOB_vt_, PIP and MAP were measured using a NICO monitor fitted with the mainstream sensor, neonatal flow adapter and a probe placed between the simulator and Y-piece of the ventilation circuit (Fig. 1). The impedance of the respiratory system (Z) was calculated basing on the total airway resistance and total compliance of the respiratory system measured by NICO.

### Statistical analysis

For the dependencies analysed in the study, Spearman correlation coefficients (R_s_) were calculated together with their P-levels. The differences between them were tested to check the influence of respiratory rate (RR) and the C_w_/C_L_ ratio on the R_s_ value. All analyses were conducted using the Statistica software (StatSoft, Inc. (2011). STATISTICA (data analysis software system), version 10; www.statsoft.com). A P-value < 0.05 was considered to be statistically significant.

## Results

**Figure 3:**
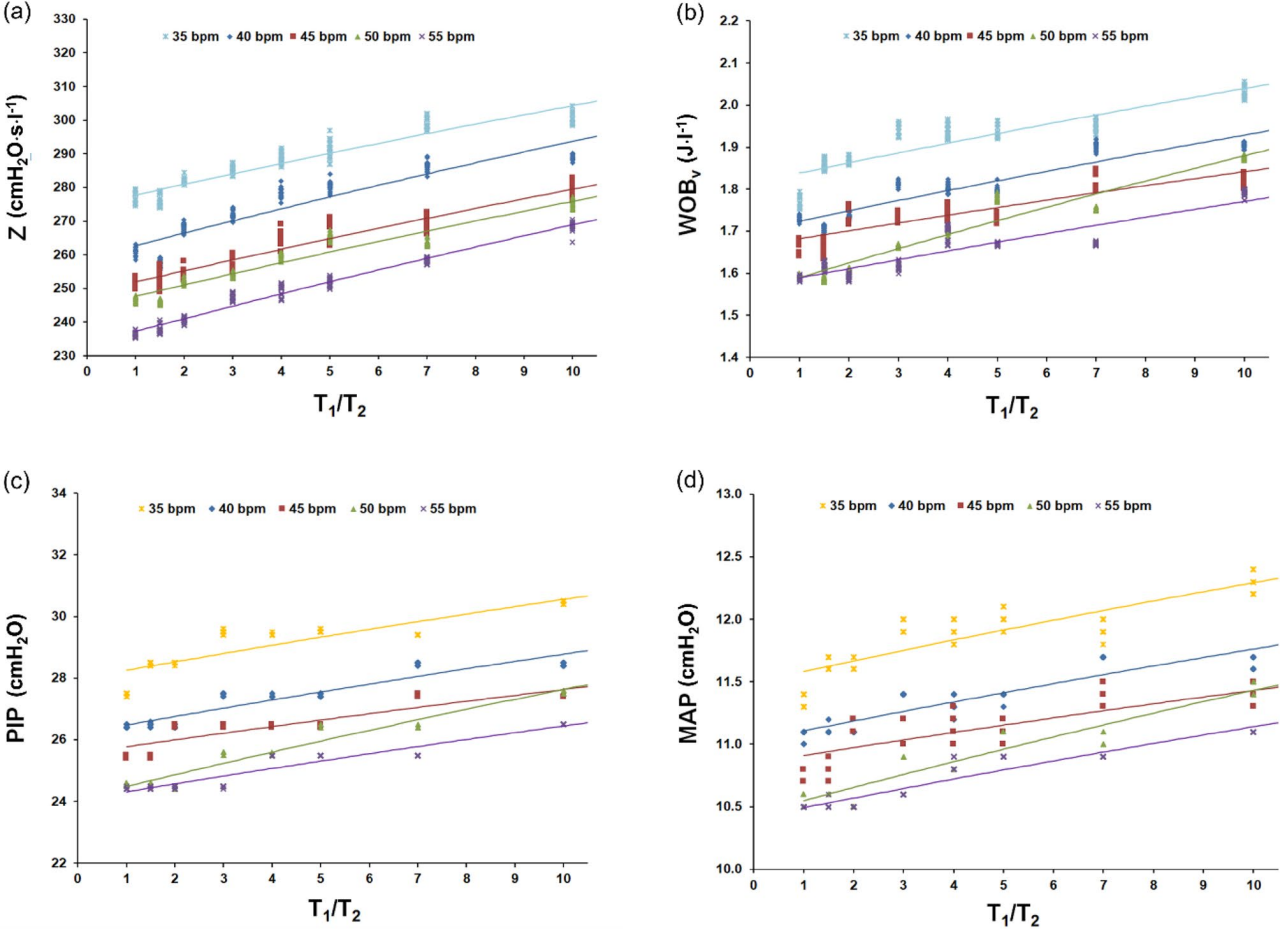
Respiratory system impedance (Z) in relation to VI degree (T_1_/T_2_) (**a**), work of breathing (WOB_vt_) vs T_1_/T_2_ (**b**), peak inspiratory pressure (PIP) vs T_1_/T_2_ (**c**), and mean airway pressure (MAP) vs T_1_/T_2_ (**d**) for patient P_3_ (R = 205 cmH_2_O·s/l; R_C_ = 131 cmH_2_O·s/l, R_1_ = R_2_ = 88 cmH_2_O s/l; C = 1.14 ml/cmH_2_O; C_w_/C_L_ = 5).

**Figure 4:**
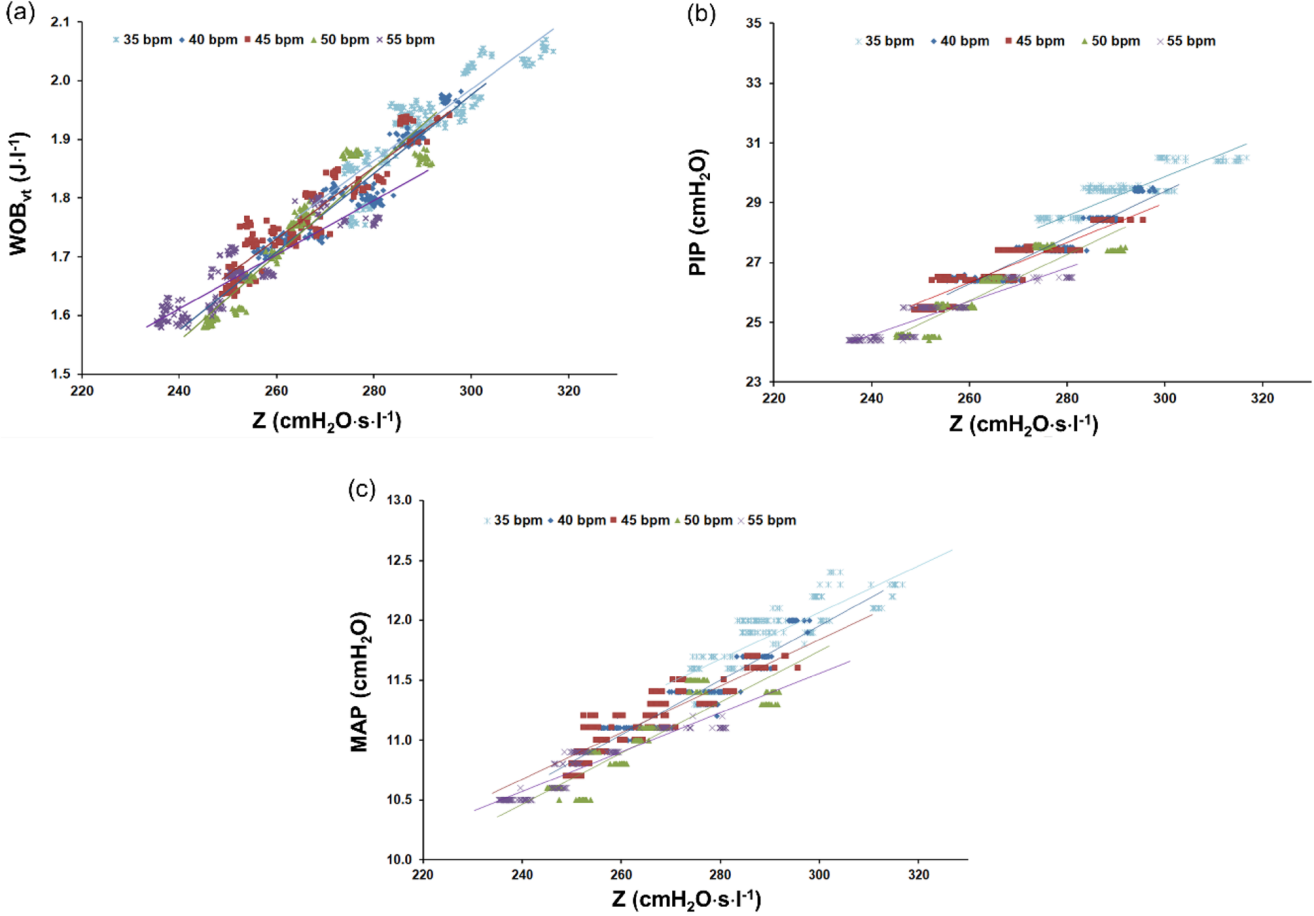
Relationship between: work of breathing (WOB_vt_) and respiratory system impedance (Z) (**a**), peak inspiratory pressure (PIP) and Z (**b**), and mean airway pressure (MAP) and Z (**c**). P_3_: R = 205 cmH_2_O·s/l, R_C_ = 131 cmH_2_O·s/l, R_1_ = R_2_ = 88 cmH_2_O·s/l, C = 1.14 ml/cmH_2_O, C_w_/C_L_ = 5.

**Figure 5:**
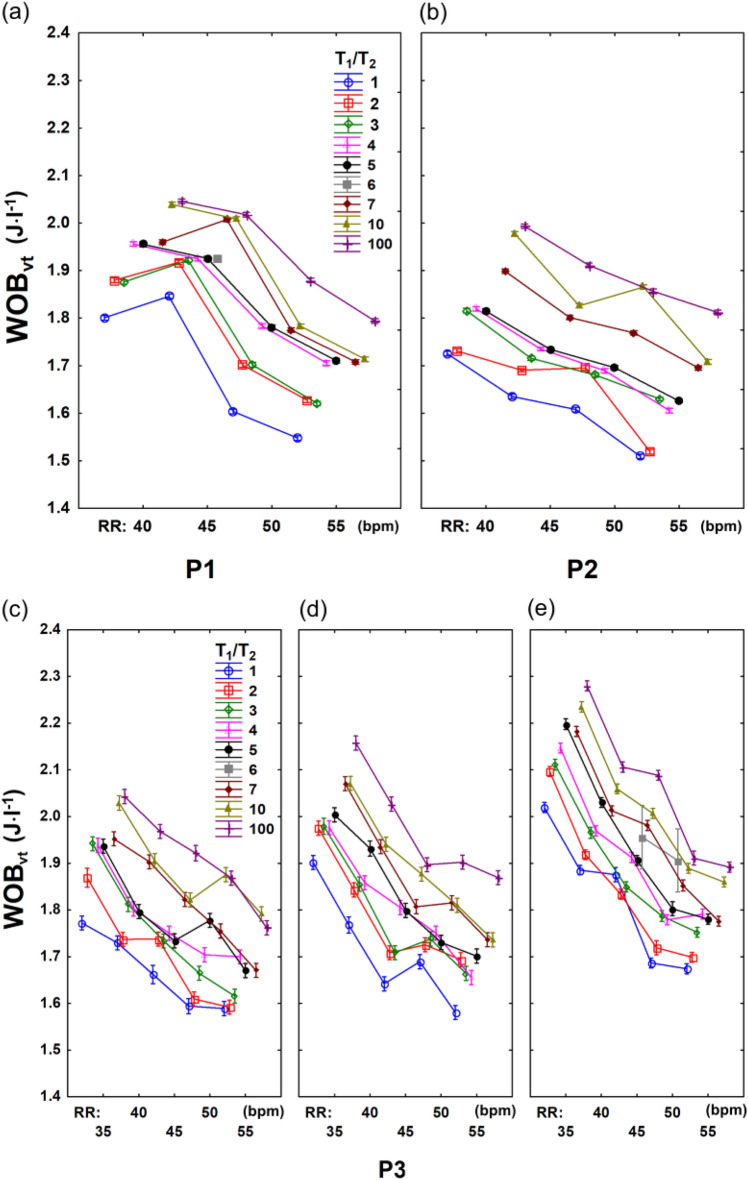
Relationship between work of breathing (WOB_vt_) and respiratory rate (RR) depending on the T_1_/T_2_ index and the C_w_/C_L_ ratio in patient P_1_ (R = 244 cmH_2_O·s/l, C = 0.8 ml/cmH_2_O, C_w_/C_L_ = 15) (**a**), P_2_ (R = 188 cmH_2_O·s/l, C = 1.26 ml/cmH_2_O, C_w_/C_L_ = 12) (**b**) and P_3_ (R = 205 cmH_2_O·s/l, C = 1.14 ml/cmH_2_O) with C_w_/C_L_ = 5 (**c**), C_w_/C_L_ = 8 (**d**), and C_w_/C_L_ = 12 (**e**).

The simulation results are presented in Figs. 3, 4 and 5.

Z, WOB_vt_, PIP and MAP as a function of the T_1_/T_2_ index, describing VI, were presented in Fig. 3. As the relationships: Z vs T_1_/T_2_, WOB_vt_ vs T_1_/T_2_, PIP vs. T_1_/T_2_, and MAP vs T_1_/T_2_ were highly influenced by the RR values (P < 0.001), separate characteristics for different RR were made. The experimental data were fitted with a second or third-degree polynomial function. The course of the mentioned characteristics was acknowledged by corresponding measurements, performed at the hypothetical value of T_1_/T_2_ = 100.

Correlation coefficients for the dependencies: Z vs T_1_/T_2_, WOB_vt_ vs T_1_/T_2_, PIP vs T_1_/T_2_, and MAP vs T_1_/T_2_ ranged from 0.7 to 0.9 (P < 0.001). For example, in the patient P_3_ (R = 205 ml/cmH_2_O, C = 1.14 ml/cmH_2_O, see Table 1) at the assumed C_w_/C_L_ = 12 and ventilated with RR = 45 bpm, correlation coefficients were as follows: 0.762 (Z vs T_1_/T_2_), 0.889 (WOB_vt_ vs. T_1_/T_2_), 0.821 (PIP vs T_1_/T_2_), 0.756 (MAP vs T_1_/T_2_), P < 0.001 (see Supplementary Tables S3–S5 online). Then, at the assumed C_w_/C_L_ = 5, correlation coefficients were as follows: 0.948 (Z vs T_1_/T_2_), 0.861 (WOB_vt_ vs T_1_/T_2_), 0.88 (PIP vs T_1_/T_2_), 0.754 (MAP vs T_1_/T_2_), P < 0.001 (Fig. 3).

Generally, the T_1_/T_2_ index is positively correlated with Z, WOB_vt_, PIP and MAP, whereas RR is negatively correlated with these ventilation indices. For example, according to our results, when ventilating a patient (R = 205 ml/cmH_2_O, C = 1.14 ml/cmH_2_O, C_w_/C_L_ = 5.3) with homogenous lungs (T_1_/T_2_ = 1) at RR = 50 bpm, the set PIP should amount about 24.4 cmH_2_O, and required WOB_vt_—1.58 J/l. However, when T_1_/T_2_ raises from 1 to 10, PIP elevates to 27.6 cmH_2_O (13%), and WOB_vt_ to 1.88 J/l (19%), (P < 0.001), i.e. a significant elevation of PIP and WOB_vt_ is forced by the increase of VI degree.

On the other hand, when changing RR from 50 to 40 bpm, in a patient (R = 205 ml/cmH_2_O, C = 1.14 ml/cmH_2_O, C_w_/C_L_ = 5) with homogenous lungs (T_1_/T_2_ = 1), PIP will increase to 26.5 cmH_2_O (8.6%), and WOB_vt_—to 1.73 J/l (9.5%), whereas in a patient with inhomogeneous lungs (T_1_/T_2_ = 10), PIP will elevate to 28.8 cmH_2_O (4.3%) and WOB_vt_ to 1.98 J/l (5.3%).

Strong and significant correlations were received for relationships between WOB_vt_ and PIP (P_1_: 0.93, P_2_: 0.95, P_3_: 0.98, P < 0.001) and WOB_vt_ vs MAP (P_1_: 0.68, P_2_: 0.95, P_3_: 0.73; P < 0.001); see Supplementary Fig. S1and Tables S1–S5 online.

The dependencies between WOB_vt_, PIP, MAP vs Z for different respiratory rates (RR) were presented in the form of characteristics in Fig. 4. They show that the WOB_vt_, PIP and MAP are highly correlated with Z. Correlation coefficients ranged from 0.7 to 0.9, e.g. in P_3_ (C_w_/C_L_ = 5) at RR = 45 bpm, there are as follows: 0.849 (WOB_vt_ vs Z), 0.833 (PIP vs Z), 0.769 (MAP vs Z), P < 0.001 (see also Supplementary Tables S1–S5 online).

Figure 5 shows the relationship between WOB_vt_ and RR depending on VI degree (T_1_/T_2_) and the C_w_/C_L_ ratio (5, 8, 12) for the patient P_3_ (R = 208 cmH_2_O s/l and C = 1.14 ml/cmH_2_O). WOB_vt_ increases with the rise of both—the T_1_/T_2_ index and C_w_/C_L_ ratio, whereas it decreases with the elevation of RR.

General Spearman correlation coefficients of Z, WOB, PIP, MAP vs T_1_/T_2_ obtained for patient P_3_ were 0.527, 0.491, 0.388 and 0.47 (P < 0.001). The coefficients increased significantly when they were calculated separately for different RR (0.828 ± 0.09; 0.722 ± 0.157; 0.621 ± 0.149; 0.792 ± 0.068), or for a given parameter pair: RR—C_w_/C_L_ (0.963 ± 0.017; 0.916 ± 0.046; 0.916 ± 0.031; 0.849 ± 0.121). Therefore, the data in Fig. 5 are presented in the form of the characteristics of RR (35, 40, 45, 50, 55 bpm) assigned to three values of C_w_/C_L_ (5, 8, 12).

## Discussion

Neonates with inhomogeneous lungs are much more vulnerable to adverse effects of ventilation and VILI than neonates with homogeneous lungs. In the cases with considerable VI, gentle, lung protective ventilation, with adequate tidal volumes and optimal PEEP as well as PIP levels, is difficult to achieve because some lung regions (having short time constants) are rapidly aerated and overdistended, whereas others, having long time constants, are insufficiently aerated and may even collapse (atelectasis)^12,13^.

The optimal PEEP and PIP should be the minimal pressure created by a ventilator at maximum inspiration and end-expiration delivered to the patient, which is required to reach acceptable levels of arterial partial pressures of O_2_ and CO_2_ and with less overdistension and less lung de-recruitment.

Evaluation of lung inhomogeneity can be accomplished using Multi Breath Washout (MBW), Electrical Impedance Tomography (EIT) or Hyperpolarized 3-Helium Magnetic Resonance Imaging (HP 3-He MRI). However, they are hardly accessible in daily clinical practice due to high costs, extensive workload and the problems with standardization^9,14–20^.

Kallio et al.^21^ evaluated the ventilation of neonates suffering from a Respiratory Distress Syndrome (RDS). They noticed that the risk of alveolar overdistention and pneumothorax in neonates with inhomogeneous lungs can be reduced by continuous monitoring of aeration and distribution of ventilation using EIT. They suggested that a steep increase of regional end-expiratory lung impedance (EELZ) rapidly signalises the risk of pneumothorax.

In another study^22^ we showed adverse phenomena taking place inside the inhomogeneous lungs of CDH infants. We observed high PIP in the more hypoplastic lung (short time constant), and high auto-PEEP in the healthier lung (long time constant) that can lead to air-trapping. The higher the difference between the time constants the higher the PIP (the risk of VILI) and the auto-PEEP.

The results of Pulletz et al.^23^, in adults with healthy lungs and ARDS, indicated that ARDS patients exhibited shorter time constants regarding the “fast and slow” regions, compared to the healthy controls. Moreover, the dorsal and ventral time constants differed and were significantly influenced by PEEP levels; both decreased with PEEP increase.

Depta, Čandik et al.^24–26^ presented a strategy based on recurrent changes in PEEP levels, enabling recruitment of lung area of long time constants and protecting the lung area of short time constants from overextension and injury. They also proposed the concept of optimal breath frequency, based on time constant measurement^24^. Their studies in ARDS patients showed that applying this technique led to a significant rise in respiratory system compliance, and improvement in CO_2_ washout, due to the increase of gas exchange area (lung recruitment)^25^, in comparison to the conventional mechanical ventilation.

However, Guevorkian et al.^27^ observed in CDH infants that respiratory system compliance (C) decreased by 30% when PEEP increased from 2 to 5 cmH_2_O. It is known, that the lower C, the higher values of PIP and MAP are required for effective ventilation. The authors indicated that hypoplastic lungs in CDH are prone to overdistension and have poor tolerance to distending pressures.

The problems with high-value local strains influencing inhomogeneous lungs were reported before by Mead^28^ and Rausch^29^. They assessed that the strain forces are much higher in inhomogeneous lungs. From the point of view of a neonatal ventilation, the ratio between chest wall compliance and lung compliance (C_w_/C_L_) is unfavourable, comparing to the older population. It amounts up to 3–6 in infants with healthy lungs^30–32^, whereas it is about 1 in healthy adults. As a result, an increase in tidal volume requires higher pressures in neonates than in adults. This fact, together with lung tissue immaturity, make neonates more vulnerable to VILI that can lead to chronic lung disease (CLD), also called bronchopulmonary dysplasia (BPD) in premature babies.

According to our knowledge, there are no clinical data on the C_w_/C_L_ ratio in CDH infants in the literature. Lungs of neonates with CDH are hypoplastic, smaller, with a reduced number of alveoli and compressed by herniated organs occupying the intrathoracic space^27^. These conditions influence respiratory system compliance (C) that can be several times lower compared to babies with healthy lungs^31–34^. Therefore, it might suggest that the C_w_/C_L_ ratio should be higher than in healthy neonatal lungs. However, our study did not prove this. On the contrary, the results suggest that the C_w_/C_L_ ratio, especially before hernia repair, can be similar or lower than in neonates with healthy lungs (3–6)^7,31–34^. It is an important issue that should be studied and explained in the future, especially in the context of the C decrease after operation. The decreased respiratory system compliance (C) is an adverse phenomenon commonly observed after hernia repair^35^*.* Currently, it is unclear why this happens. Probably, the herniated organs occupying the thorax before hernia repair, compress not only the lungs, but also push the chest wall outside, limiting the thorax movements and making its muscles stiffer (C_w_↓). The distribution of the forces acting within the thorax changes, after the herniated organs are removed from the thoracic to the abdominal cavity. These forces, resulting from the direct pressure of the herniated organs on the lungs and chest wall, stop to act. On the other hand, they may exert strain on the diaphragm from the bottom, indirectly affecting the lungs and the chest wall; however, this influence is limited by the physical barrier created by the diaphragm. The effect may be variable, depending on many factors, such as the initial thorax content (stomach, liver, intestines), the properties of the diaphragmatic muscle itself (anatomy, saturation, the size and exact location of the hole), the method of hernia repairing (stitching or patching with artificial material, e.g. Gore-Tex).

It is known, that liver herniation significantly increases the risk of mortality in CDH infants^3,7,34,35^. The higher percentage of the liver in the thorax (%LH), the higher the risk of therapy failure. Probably, the higher the %LH, the greater decline of C as a result of the operation can be expected. Sakai^35^ showed that a higher than 50% decrease in C was associated with mortality. According to Takayasu et al.^36^, who enrolled 49 children treated for CDH within two decades in one institution, liver-up and patch repair were the main risk factors for long-term complications (it concerned 50% of the patients with liver-up and 36% with patch-repair). In the study conducted by Brandt et al.^37^, covering the treatment of 66 CDH infants within 16 years, a liver-up position was found in 51% of survivors as opposed to 80% of non-survivors (P < 0.05)^38^.

Jancelewicz et al.^7^, based on their long-term cohort study, observed that the patch repair was the most strongly and independently associated parameter with subsequent surgical complications. As shown by Lally et al.^38^, large hernia size (that requires artificial patch repair) strongly reduces overall survival as well as increases the risk of multiple adverse outcomes.

CDH neonates belong to one of the most complex and demanding patients treated in NICU/PICU (Neonatal/ Paediatric Intensive Care Units). According to the last CDH Euro Consortium and APSA OEBP committee^27^ gentle ventilation with limited PIP up to 25 ± 2 cmH_2_O, PEEP of 3–5 cmH_2_O, with permissive hypercapnia strategy^6^ is recommended during ventilation of CDH neonates. As a first-line strategy CMV is preferred over HFV.

The results of our study simulating PCV mode in CDH newborns seem to indicate that strong relationships exist between the ratio of time constants of the right and left lung (T_1_/T_2_) and ventilation parameters: WOB_vt_, PIP, and MAP. Increased T_1_/T_2_ results in increase in respiratory system impedance (Z), work of breathing (WOB_vt_) and the airway pressures (PIP and MAP) in the patient-ventilator system. All the above indicate that the T_1_/T_2_ index can serve as a lung inhomogeneity measure. Secondly, this index could probably be useful in the prediction of optimal PIP and MAP values at the bedside, corresponding to the degree of lung ventilation inhomogeneity. Thirdly, in selected cases, the use of the above measurements could possibly help to decide on changing ventilation strategy from CMV to HFV; for example using algorithms similar to that proposed by us^9^. However, further extensive studies are necessary.

### Potential clinical implementation of the method in the future

**Figure 6:**
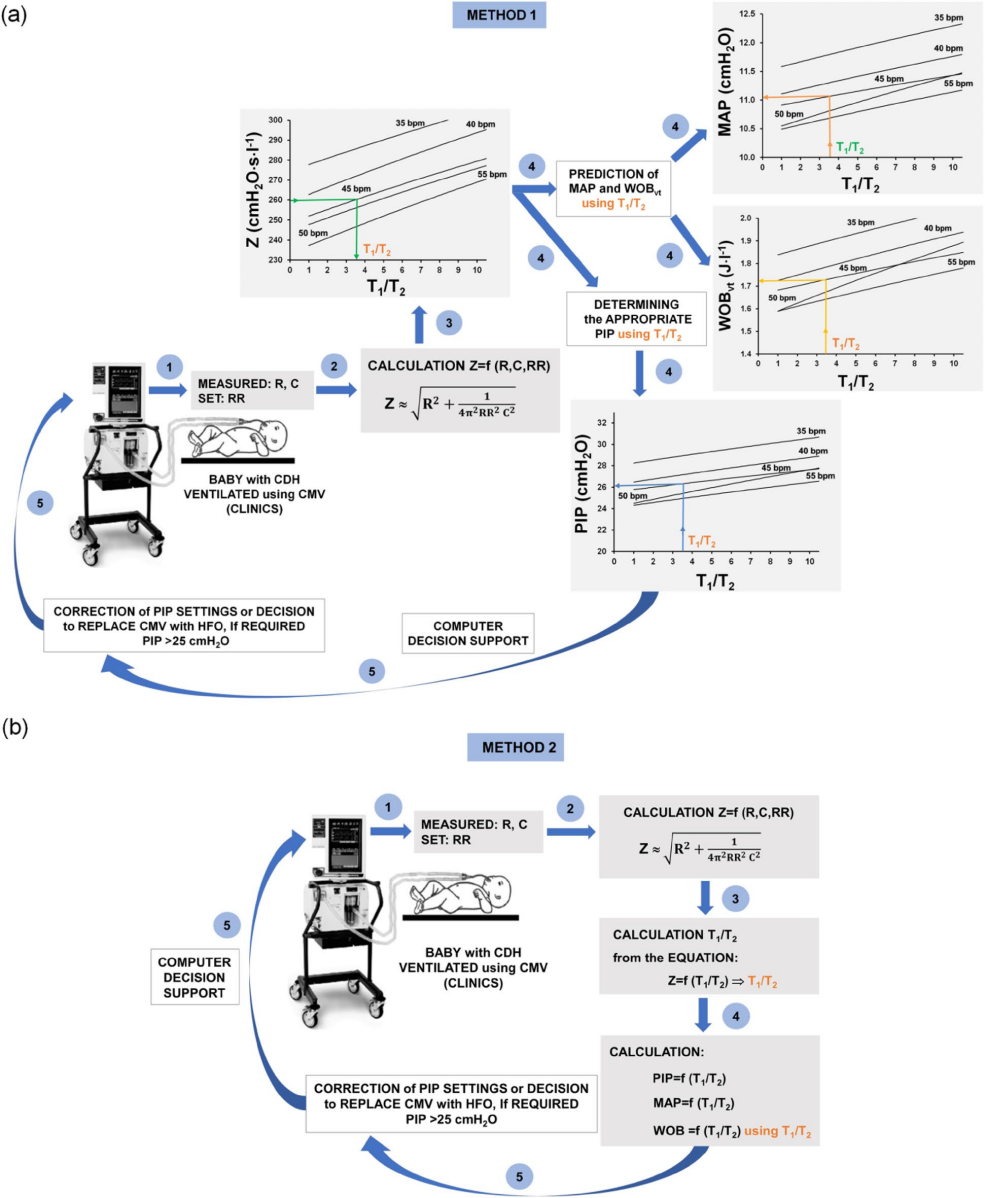
Examples of clinical application of the VI assessment using T_1_/T_2_ index to correct settings of PIP at PCV mode of conventional mechanical ventilation (CMV): method 1 (**a)**, method 2 (**b**). Inertance (L) was abandoned in the calculation of impedance (step 2) for simplicity; its significance is low at RR < 60 bpm (< 1 Hz).

Below, we present graphically how our method could possibly be used in clinical practice. Figure 6 shows two examples of the clinical application of the VI assessment based on the T_1_/T_2_ index at the bedside with the use of respiratory parameters available from the ventilator connected to the patient’s respiratory system. First, at the set RR, the R and C measured by the ventilator are found (step 1). Then, using R, C and RR, respiratory system impedance Z is calculated (step 2). Using the Z value, the T_1_/T_2_ value is obtained for set RR from the dependence Z = f (T_1_/T_2_), in a graphical way (Fig. 6a) or solving the equation Z = f (T_1_/T_2_) (Fig. 6b) (step 3). Based on the T_1_/T_2_ value, PIP, MAP and WOB_vt_ are assessed, graphically (Fig. 6a) or using relevant equations MAP = f (T_1_/T_2_) and WOB_vt_ = f (T_1_/T_2_) (Fig. 6b) (step 4). As a result, ventilator settings are adjusted to the VI degree (T_1_/T_2_), i.e. adequate PIP is set on the ventilator. If predicted PIP is higher than recommended in CDH, the early, elective change to HFO can be made (step 5).

CDH is a rare neonatal disease, and the collection of a large group of patients is a long-term task, but we are in the course of acquiring more data. Another option we might consider is to conduct an animal model study. However, performing a prospective study in an animal model depends on many factors (economic costs, ethical approval, implementation of an animal CDH-model, etc.). The difficulties in transforming animal study results into a clinical method implementation in humans should be considered carefully. From our point of view, laboratory simulations based on retrospective clinical data taken from CDH neonates seem to be a better solution, although analyzing data from more patients is indispensable. It is warranted to create more data within laboratory research prior to the implementation of the method in the daily practice.

### Limitations

The simulations were based on some theoretical assumptions that would require verification in the future investigations. The assumptions concerned value distribution of lung model parameters, between central and peripheral airways (C_w_/C_L_, R_c_/R). Clinical data on the topic are hardly available in the literature^32,33^. Based on our study results, we hypothesize that in CDH infants, before hernia repair, the chest wall is stiffer than in healthy infants. It means that the C_w_/C_L_ ratio may be lower than it has been believed, so far. Further studies are necessary to prove actual C_w_/C_L_ relation, to explain the reasons for decreased compliance C after hernia repair and to move the research from the simulation level to the clinical use.

## Summary

The main study results confirm the pilot study results^9^ and indicate the following:

- there are strong and significant correlations between respiratory system impedance (Z), ventilation parameters (WOB_vt_, PIP, MAP) and ventilation inhomogeneity degree, described by the T_1_/T_2_ index (0.8–0.9, P < 0.001); the correlations are dependent on respiratory rate (RR) and the ratio between chest wall compliance and lung compliance (C_W_/C_L_); these dependencies can be expressed in the form of characteristics consisted of several curves of polynomial functions (Fig. 3, Supplementary Tables S1–S5 online);
- the work of breathing (WOB_vt_), PIP, and MAP highly correlate with respiratory system impedance (Z) (0.8–09, P < 0.001) (Fig. 4); the correlation is nearly-linear and dependent on respiratory rate (RR) and the ratio between chest wall compliance and lung compliance (C_W_/C_L_) (Fig. 5, Supplementary Tables S1-S5 online);
- the work of breathing (WOB_vt_) highly correlates with PIP and MAP (0.9, P < 0.001), in the patient-ventilator system; the correlation is linear and independent of RR and the C_W_/C_L_ ratio (Supplementary Fig. S1 online).

These results may indicate that the T_1_/T_2_ index could potentially serve as a measure of ventilation inhomogeneity degree in the future. It could be clinically useful to optimize ventilation settings based on objective, measurable parameters, i.e. to determine appropriate levels of PIP and MAP and choose the correct strategy of ventilation. If the predicted PIP and MAP appeared to be too high to continue CMV, due to the risk of lung injury, HFV might be implemented earlier compared to the existing procedures.

## Supplementary Information

Supplementary Information.

## Data Availability

The datasets used and/ or analyzed in the study are available from the corresponding author for reasonable request.
