# Supplementary material for: A new method of ventilation inhomogeneity assessment based on a simulation study using clinical data on congenital diaphragmatic hernia cases
Source: Sci Rep. 2022 Dec 31;12:22635. doi: 10.1038/s41598-022-27027-8 (PMC9805438; doi:10.1038/s41598-022-27027-8)
Supplement: Supplementary file 1 — Supplementary Information. [file 41598_2022_27027_MOESM1_ESM.docx]

Supplementary Information

A new method of ventilation inhomogeneity assessment based on a simulation study using clinical data on congenital diaphragmatic hernia cases

Barbara Stankiewicz, Magdalena Mierzewska-Schmidt, Krzysztof Jakub Pałko, Artur Baranowski, Marek Darowski, Maciej Kozarski

**Table S1.** The parameters of regression function, determination and correlation coefficients obtained for patient P_1_

| Relationship | RR (bpm) | The parameters of function  (a·x^3^+b·x^2^ + c·x + d) | | | | R^2^ | R_s_ |
| --- | --- | --- | --- | --- | --- | --- | --- |
|  |  | a | b | c | d |  |  |
| Z  vs  T_1_/T_2_ | 40 | - | -0.0103 | 1.232 | 358 | 0.695 | 0.965 |
|  | 45 | - | -0.0156 | 1.839 | 326 | 0.8 | 0.92 |
|  | 50 | - | -0.0161 | 1.929 | 308 | 0.893 | 0.991 |
|  | 55 | - | -0.0155 | 1.847 | 291 | 0.926 | 0.969 |
|  | 60 | - | -0.0174 | 2.048 | 286 | 0.743 | 0.946 |
| WOB  vs  T_1_/T_2_ | 40 | 1·10^-5^ | -0.0014 | 0.035 | 1.8 | 0.906 | 0.949 |
|  | 45 | 9·10^-6^ | -0.0012 | 0.031 | 1.82 | 0.878 | 0.9 |
|  | 50 | 1·10^-5^ | -0.0013 | 0.029 | 1.64 | 0.822 | 0.88 |
|  | 55 | 1·10^-5^ | -0.0013 | 0.028 | 1.57 | 0.819 | 0.875 |
|  | 60 | 1·10^-5^ | -0.0018 | 0.04 | 1.45 | 0.917 | 0.856 |
| PIP  vs  T_1_/T_2_ | 40 | 1⋅10^-4^ | -0.017 | 0.43 | 27.7 | 0.898 | 0.947 |
|  | 45 | 1⋅10^-4^ | -0.0154 | 0.367 | 27.4 | 0.85 | 0.873 |
|  | 50 | 1⋅10^-4^ | -0.0163 | 0.356 | 24.9 | 0.841 | 0.925 |
|  | 55 | 1⋅10^-4^ | -0.0159 | 0.342 | 24 | 0.773 | 0.833 |
|  | 60 | 2⋅10^-4^ | -0.0246 | 0.532 | 22 | 0.923 | 0.806 |
| MAP  vs  T_1_/T_2_ | 40 | 3·10^-5^ | -0.0041 | 0.102 | 10.13 | 0.876 | 0.942 |
|  | 45 | 3·10^-5^ | -0.0042 | 0.094 | 9.37 | 0.76 | 0.867 |
|  | 50 | 3·10^-5^ | -0.0043 | 0.097 | 9.36 | 0.845 | 0.951 |
|  | 55 | 3·10^-5^ | -0.0041 | 0.086 | 9.13 | 0.652 | 0.837 |
|  | 60 | 7·10^-5^ | -0.0084 | 0.17 | 8.68 | 0.934 | 0.808 |
| WOB vs PIP | 40-60 | - | - | 0.069 | -0.069 | 0.93 | 0.983 |
| WOB vs MAP | 40-60 | - | - | 0.264 | -0.791 | 0.679 | 0.868 |
| WOB vs Z | 40 |  |  | 0.008 | -1.116 | 0.662 | 0.913 |
|  | 45 |  |  | 0.005 | 0.211 | 0.683 | 0.808 |
|  | 50 |  |  | 0.006 | -0.112 | 0.8 | 0.872 |
|  | 55 |  |  | 0.005 | 0.094 | 0.67 | 0.814 |
|  | 60 |  |  | 0.007 | 0.369 | 0.913 | 0.9 |
| PIP vs Z | 40 |  |  | 0.097 | -5.9 | 0.62 | 0.89 |
|  | 45 |  |  | 0.058 | 9.5 | 0.632 | 0.789 |
|  | 50 |  |  | 0.065 | 5.5 | 0.783 | 0.917 |
|  | 55 |  |  | 0.059 | 7.4 | 0.602 | 0.79 |
|  | 60 |  |  | 0.082 | -0,3 | 0.842 | 0.796 |
| MAP vs Z | 40 | - | 0.0007 | -0.485 | 94.7 | 0.627 | 0.883 |
|  | 45 | -3⋅10^-5^ | 0.0282 | -9.415 | 1056 | 0.553 | 0.75 |
|  | 50 | 5⋅10^-5^ | -0.0478 | 15.493 | 1669 | 0.909 | 0.943 |
|  | 55 | 1 ⋅10^-4^ | -0.0907 | 27.863 | 2842 | 0.694 | 0.79 |
|  | 60 | 4⋅10^-5^ | -0.0384 | 11.551 | 1150 | 0.821 | 0.795 |
| Z - respiratory system impedance, WOB – work of breathing, PIP – peak inspiratory pressure, MAP – mean airway pressure. RR – respiratory rate, R^2^ - coefficient of determination and R_s_ – Spearman correlation coefficient with P<0.001. | | | | | | | |

**Table S2.** The parameters of regression function, determination and correlation coefficients obtained in patient P2.

| Relationship | RR (bpm) | The parameters of function  (a·x^3^+b·x^2^ + c·x + d) | | | | R^2^ | R_s_ |
| --- | --- | --- | --- | --- | --- | --- | --- |
|  |  | a | b | c | d |  |  |
| Z vs T_1_/T_2_ | 40 | 0.0016 | -0.205 | 4.77 | 257 | 0.961 | 0.967 |
|  | 45 | 0.0013 | -0.167 | 4.51 | 241 | 0.959 | 0.969 |
|  | 50 | 0.0013 | -0.167 | 4.43 | 237 | 0.954 | 0.985 |
|  | 55 | 0.0016 | -0.212 | 5.41 | 227 | 0.954 | 0.982 |
|  | 60 | 0.0012 | -0.158 | 4.11 | 221 | 0.95 | 0.969 |
| WOB_vt_ vs T_1_/T_2_ | 40 | 2⋅10^-5^ | -0.002 | 0.048 | 1.657 | 0.952 | 0.936 |
|  | 45 | 1⋅10^-5^ | -0.0012 | 0.035 | 1.606 | 0.915 | 0.909 |
|  | 50 | 1⋅10^-5^ | -0.0017 | 0.043 | 1.559 | 0.923 | 0.895 |
|  | 55 | 1⋅10^-5^ | -0.0011 | 0.039 | 1.482 | 0.885 | 0.891 |
|  | 60 | 1⋅10^-5^ | -0.0015 | 0.03 | 1.508 | 0.892 | 0.905 |
| PIP vs T_1_/T_2_ | 40 | 0.0002 | -0.0212 | 0.544 | 26.8 | 0.949 | 0.923 |
|  | 45 | 0.0001 | -0.0137 | 0.403 | 26 | 0.905 | 0.82 |
|  | 50 | 0.0001 | -0.0182 | 0.482 | 25.1 | 0.919 | 0.952 |
|  | 55 | 0.0001 | -0.0141 | 0.411 | 24.1 | 0.897 | 0.905 |
|  | 60 | 0.0001 | -0.0176 | 0.434 | 24 | 0.898 | 0.896 |
| MAP  vs.  T_1_/T_2_ | 40 | 4⋅10^-5^ | -0.0056 | 0.143 | 11.3 | 0.941 | 0.954 |
|  | 45 | 2⋅10^-5^ | -0.0033 | 0.1 | 11.2 | 0.82 | 0.826 |
|  | 50 | 3⋅10^-5^ | -0.0045 | 0.12 | 10.9 | 0.924 | 0.978 |
|  | 55 | 3⋅10^-5^ | -0.0036 | 0.108 | 10.8 | 0.861 | 0.907 |
|  | 60 | 4⋅10^-5^ | -0.0048 | 0.124 | 10.7 | 0.877 | 0.904 |
| WOB vs PIP | 40-60 | - | - | 0.073 | -0.246 | 0.952 | 0.958 |
| WOB vs MAP | 40-60 | - | - | 0.303 | -1.758 | 0.945 | 0.961 |
| WOB vs Z | 40 | - | - | 0.01 | -0.739 | 0.877 | 0.937 |
|  | 45 | - | - | 0.008 | -0.306 | 0.891 | 0.913 |
|  | 50 | - | - | 0.008 | -0.336 | 0.837 | 0.889 |
|  | 55 | - | - | 0.007 | -0.111 | 0.856 | 0.888 |
|  | 60 |  |  | 0.006 | 0.256 | 0.727 | 0.889 |
| PIP vs Z | 40 | - | - | 0.1062 | -0.3 | 0.824 | 0.908 |
|  | 45 | - | - | 0.0884 | 4.8 | 0.825 | 0.76 |
|  | 50 | - | - | 0.0922 | 3.5 | 0.813 | 0.928 |
|  | 55 | - | - | 0.0756 | 7.1 | 0.805 | 0.894 |
|  | 60 | - | - | 0.0887 | 4.6 | 0.828 | 0.866 |
| MAP vs Z | 40 | - | - | 0.0281 | 4.1 | 0.827 | 0.93 |
|  | 45 | - | - | 0.0232 | 5.6 | 0.784 | 0.807 |
|  | 50 | - | - | 0.0242 | 5.3 | 0.82 | 0.961 |
|  | 55 | - | - | 0.0199 | 6.3 | 0.763 | 0.892 |
|  | 60 | - | - | 0.0254 | 5.2 | 0.744 | 0.874 |
| Z - respiratory system impedance, WOB – work of breathing, PIP – peak inspiratory pressure, MAP – mean airway pressure. RR – respiratory rate, R^2^ - coefficient of determination with P<0.001 | | | | | | | |

**Table S3** The parameters of regression function, determination and Spearman correlation coefficients obtained for patient P_3_^a^.

| Relationship | RR (bpm) | The parameters of function  (a·x^3^+b·x^2^ + c·x + d) | | | | R^2^ | R_s_ |
| --- | --- | --- | --- | --- | --- | --- | --- |
|  |  | a | b | c | d |  |  |
| Z^a^  vs  T_1_/T_2_ | 35 | - | -0.029 | 3.272 | 275 | 0.942 | 0.967 |
|  | 40 | - | -0.035 | 3.831 | 259 | 0.899 | 0.974 |
|  | 45 | - | -0.03 | 3.394 | 249 | 0.948 | 0.967 |
|  | 50 | - | -0.03 | 3.465 | 244 | 0.962 | 0.966 |
|  | 55 | - | -0.035 | 3.9 | 233 | 0.982 | 0.986 |
| WOB  vs  T_1_/T_2_ | 35 | - | -0.0002 | 0.025 | 1.814 | 0.817 | 0.915 |
|  | 40 | - | -0.0002 | 0.025 | 1.7 | 0.914 | 0.908 |
|  | 45 | - | -0.0002 | 0.02 | 1.663 | 0.861 | 0.883 |
|  | 50 | - | -0.0003 | 0.036 | 1.554 | 0.949 | 0.95 |
|  | 55 | - | -0.0002 | 0.023 | 1.567 | 0.862 | 0.879 |
| PIP  vs.  T_1_/T_2_ | 35 | - | -0.0026 | 0.284 | 28 | 0.798 | 0.87 |
|  | 40 | - | -0.0025 | 0.283 | 26.2 | 0.923 | 0.911 |
|  | 45 | - | -0.002 | 0.229 | 25.6 | 0.88 | 0.877 |
|  | 50 | - | -0.0036 | 0.389 | 24.1 | 0.943 | 0.915 |
|  | 55 | - | -0.0024 | 0.264 | 24.1 | 0.926 | 0.954 |
| MAP  vs  T_1_/T_2_ | 35 | - | -0.0008 | 0.088 | 11.5 | 0.75 | 0.895 |
|  | 40 | - | -0.0007 | 0.08 | 11 | 0.913 | 0.943 |
|  | 45 | - | -0.0006 | 0.064 | 10.9 | 0.754 | 0.858 |
|  | 50 | - | -0.001 | 0.109 | 10.4 | 0.895 | 0.9 |
|  | 55 | - | -0.0007 | 0.08 | 10.4 | 0.927 | 0.968 |
| WOB vs PIP | 35-55 | - | - | 0.07 | -0.11 | 0.976 | 0.97 |
| WOB vs MAP | 35-55 | - | - | 0.238 | -0.854 | 0.727 | 0.971 |
| WOB vs Z | 35 | - | - | 0.006 | 0.158 | 0.782 | 0.9 |
|  | 40 | - | - | 0.007 | -0.026 | 0.897 | 0.927 |
|  | 45 | - | - | 0.006 | 0.14 | 0.849 | 0.889 |
|  | 50 | - | - | 0.007 | -0.213 | 0.891 | 0.972 |
|  | 55 | - | - | 0.005 | 0.51 | 0.813 | 0.874 |
| PIP vs Z | 35 | - | - | 0.065 | 10.3 | 0.721 | 0.821 |
|  | 40 | - | - | 0.078 | 6.1 | 0.879 | 0.886 |
|  | 45 | - | - | 0.067 | 9 | 0.833 | 0.854 |
|  | 50 | - | - | 0.077 | 5.7 | 0.848 | 0.922 |
|  | 55 | - | - | 0.056 | 11.1 | 0.876 | 0.94 |
| MAP vs Z | 35 | - | - | 0.02 | 6.23 | 0.678 | 0.863 |
|  | 40 | - | - | 0.023 | 5.13 | 0.86 | 0.935 |
|  | 45 | - | - | 0.019 | 6.02 | 0.769 | 0.861 |
|  | 50 | - | - | 0.021 | 5.36 | 0.791 | 0.908 |
|  | 55 | - | - | 0.016 | 6.63 | 0.869 | 0.957 |
| Z - respiratory system impedance, WOB – work of breathing, PIP – peak inspiratory pressure, MAP – mean airway pressure. RR – respiratory rate, R^2^ - coefficient of determination and R_s_ - Spearman correlation coefficient with P<0.001. ^a^C_W_/C_L_=5. | | | | | | | |

**Table S4** The parameters of regression function, determination and Spearman correlation coefficients obtained for patient P_3_^a^.

| Relationship | RR (bpm) | The parameters of function  (a·x^3^+b·x^2^ + c·x + d) | | | | R^2^ | R_s_ |
| --- | --- | --- | --- | --- | --- | --- | --- |
|  |  | a | b | c | d |  |  |
| Z  vs  T_1_/T_2_ | 35 | - | -0.0265 | 2.977 | 297 | 0.912 | 0.968 |
|  | 40 | - | -0.033 | 3.635 | 279 | 0.94 | 0.973 |
|  | 45 | - | -0.022 | 2.517 | 271 | 0.882 | 0.962 |
|  | 50 | - | -0.028 | 3.156 | 253 | 0.938 | 0.942 |
|  | 55 | - | -0.016 | 1.935 | 253 | 0.834 | 0.96 |
| WOB  vs  T_1_/T_2_ | 35 | - | -0.0002 | 0.023 | 1.964 | 0.89 | 0.948 |
|  | 40 | - | -0.0002 | 0.023 | 1.838 | 0.911 | 0.972 |
|  | 45 | - | -0.0002 | 0.02 | 1.79 | 0.889 | 0.924 |
|  | 50 | - | -0.0002 | 0.021 | 1.651 | 0.843 | 0.909 |
|  | 55 | - | -0.0001 | 0.012 | 1.693 | 0.707 | 0.91 |
| PIP  vs  T_1_/T_2_ | 35 | - | -0.0025 | 0.276 | 30.6 | 0.864 | 0.958 |
|  | 40 | - | -0.0021 | 0.241 | 28.6 | 0.889 | 0.947 |
|  | 45 | - | -0.0019 | 0.215 | 27.5 | 0.821 | 0.925 |
|  | 50 | - | -0.0022 | 0.243 | 25.7 | 0.841 | 0.872 |
|  | 55 | - | -0.0013 | 0.152 | 25.8 | 0.716 | 0.923 |
| MAP  vs  T_1_/T_2_ | 35 | - | -0.0008 | 0.085 | 12.3 | 0.867 | 0.949 |
|  | 40 | - | -0.0006 | 0.072 | 11.7 | 0.872 | 0.968 |
|  | 45 |  | -0.0005 | 0.061 | 11.6 | 0.756 | 0.908 |
|  | 50 |  | -0.0006 | 0.072 | 10.9 | 0.848 | 0.853 |
|  | 55 |  | -0.0004 | 0.041 | 10.9 | 0.634 | 0.945 |
| WOB vs PIP | 35-55 | - | - | 0.07 | -0.11 | 0.976 | 0.972 |
| WOB vs MAP | 35-55 | - | - | 0.238 | -0.854 | 0.727 | 0.98 |
| WOB vs Z | 35 | - | - | 0.0079 | -0.368 | 0.887 | 0.934 |
|  | 40 | - | - | 0.0067 | -0.03 | 0.891 | 0.951 |
|  | 45 | - | - | 0.0076 | -0.259 | 0.909 | 0.921 |
|  | 50 | - | - | 0.0068 | -0.077 | 0.873 | 0.867 |
|  | 55 | - | - | 0.0059 | 0.192 | 0.833 | 0.854 |
| PIP vs Z | 35 | - | - | 0.091 | 3.6 | 0.861 | 0.918 |
|  | 40 | - | - | 0. 071 | 8.7 | 0.814 | 0.9 |
|  | 45 | - | - | 0.081 | 5.8 | 0.802 | 0.885 |
|  | 50 | - | - | 0.073 | 7.4 | 0.823 | 0.77 |
|  | 55 | - | - | 0.07 | 8.2 | 0.856 | 0.824 |
| MAP vs Z | 35 | - | - | 0.027 | 4.2 | 0.875 | 0.918 |
|  | 40 | - | - | 0.02 | 6.1 | 0.818 | 0.927 |
|  | 45 | - | - | 0.023 | 5.3 | 0.748 | 0.895 |
|  | 50 | - | - | 0.022 | 5.5 | 0.829 | 0.754 |
|  | 55 | - | - | 0.019 | 6.2 | 0.77 | 0.871 |
| Z - respiratory system impedance, WOB – work of breathing, PIP – peak inspiratory pressure, MAP – mean airway pressure. RR – respiratory rate, R^2^ - coefficient of determination with P<0.001. ^a^C_W_/C_L_=8. | | | | | | | |

**Table S5** The parameters of regression function, determination and Spearman correlation coefficients obtained for patient P_3_^a^.

| Relationship | RR (bpm) | The parameters of function (a·x^3^+b·x^2^ + c·x + d) | | | | R^2^ | R_s_ |
| --- | --- | --- | --- | --- | --- | --- | --- |
|  |  | a | b | c | d |  |  |
| Z  vs  T_1_/T_2_ | 35 | - | -0.0274 | 3.07 | 289 | 0.922 | 0.959 |
|  | 40 | - | -0.0287 | 3.26 | 270 | 0.93 | 0.977 |
|  | 45 | - | -0.277 | 3.16 | 258 | 0.931 | 0.912 |
|  | 50 | - | -0.0284 | 3.16 | 251 | 0.926 | 0.971 |
|  | 55 | - | -0.0231 | 2.71 | 243 | 0.935 | 0.964 |
| WOB  vs  T_1_/T_2_ | 35 | - | -0.0002 | 0.022 | 1.9 | 0.919 | 0.946 |
|  | 40 | - | -0.0002 | 0.021 | 1.8 | 0.885 | 0.96 |
|  | 45 | - | -0.0002 | 0.025 | 1.7 | 0.863 | 0.9 |
|  | 50 | - | -0.0001 | 0.017 | 1.7 | 0.902 | 0.946 |
|  | 55 | - | -0.0001 | 0.017 | 1.6 | 0.871 | 0.785 |
| PIP  vs  T_1_/T_2_ | 35 | - | -0.0022 | 0.25 | 29.5 | 0.901 | 0.947 |
|  | 40 | - | -0.0023 | 0.261 | 27.7 | 0.861 | 0.934 |
|  | 45 | - | -0.0027 | 0.302 | 25.8 | 0.882 | 0.886 |
|  | 50 | - | -0.0022 | 0.246 | 25.6 | 0.892 | 0.933 |
|  | 55 | - | 0.002 | 0.231 | 24.6 | 0.894 | 0.882 |
| MAP  vs  T_1_/T_2_ | 35 | - | -0.0007 | 0.077 | 11.9 | 0.898 | 0.748 |
|  | 40 | - | -0.0008 | 0.085 | 11.4 | 0.89 | 0.639 |
|  | 45 | - | -0.0007 | 0.082 | 10.9 | 0.834 | 0.641 |
|  | 50 | - | -0.0006 | 0.072 | 10.9 | 0.85 | 0.877 |
|  | 55 | - | -0.0005 | 0.062 | 10.6 | 0.871 | 0.641 |
| WOB vs PIP | 35-55 | - | - | 0.07 | -0.11 | 0.976 | 0.979 |
| WOB vs MAP | 35-55 | - | - | 0.238 | -0.854 | 0.727 | 0.834 |
| WOB vs. Z | 35 | - | - | 0.0071 | -0.155 | 0.885 | 0.952 |
|  | 40 | - | - | 0.0062 | 0.124 | 0.909 | 0.957 |
|  | 45 | - | - | 0.0065 | -0.013 | 0.866 | 0.838 |
|  | 50 | - | - | 0.0061 | 0.145 | 0.826 | 0.9 |
|  | 55 | - | - | 0.0065 | 0.032 | 0.863 | 0.827 |
| PIP vs Z | 35 | - | - | 0.081 | 6.23 | 0.831 | 0.897 |
|  | 40 | - | - | 0.073 | 8.06 | 0.863 | 0.905 |
|  | 45 | - | - | 0.072 | 7.51 | 0.803 | 0.742 |
|  | 50 | - | - | 0.079 | 5.83 | 0.783 | 0.867 |
|  | 55 | - | - | 0.07 | 7.86 | 0.815 | 0.883 |
| MAP vs Z | 35 | - | - | 0.024 | 5.02 | 0.844 | 0.932 |
|  | 40 | - | - | 0.023 | 5.35 | 0.867 | 0.919 |
|  | 45 | - | - | 0.021 | 5.46 | 0.804 | 0.73 |
|  | 50 | - | - | 0.023 | 5.08 | 0.722 | 0.9 |
|  | 55 | - | - | 0.019 | 6 | 0.796 | 0.867 |
| Z - respiratory system impedance, WOB – work of breathing, PIP – peak inspiratory pressure, MAP – mean airway pressure. RR – respiratory rate, R^2^ - coefficient of determination with P<0.001.b ^a^C_W_/C_L_=12 | | | | | | | |

**Table S6** Spearman correlation coefficients for dependences between respiratory parameters and ventilation inhomogeneity degree in patient P_3_^a^.

| Z vs T_1_/T_1_ | RR (bmp) | C_w_/C_L_ | Z(RR) vs T_1_/T_1_ | RR (bmp) | C_w_/C_L_ | Z(RR,C_W_/C_L_) vs T_1_/T_1_ |
| --- | --- | --- | --- | --- | --- | --- |
| 0.527 | 35 | 8.3±2.1 | 0.683 | 35 | 5 | 0.967 |
|  |  |  |  | 40 |  | 0.974 |
|  |  |  |  | 45 |  | 0.967 |
|  | 40 |  | 0.815 | 50 |  | 0.966 |
|  |  |  |  | 55 |  | 0.986 |
|  |  |  |  | 35 | 8 | 0.968 |
|  | 45 |  | 0.841 | 40 |  | 0.973 |
|  |  |  |  | 45 |  | 0.962 |
|  |  |  |  | 50 |  | 0.942 |
|  | 50 |  | 0.922 | 55 |  | 0.96 |
|  |  |  |  | 35 | 12 | 0.959 |
|  |  |  |  | 40 |  | 0.977 |
|  | 55 |  | 0.878 | 45 |  | 0.912 |
|  |  |  |  | 50 |  | 0.971 |
|  |  |  |  | 55 |  | 0.964 |
|  | Mean±SD: | | 0.828±0.09* | Mean±SD: | | 0.963±0.017*^,^* |
| WOB_vt_ vs T_1_/T_1_ | RR (bmp) | C_w_/C_L_ | WOB_vt_(RR)  vs T_1_/T_1_ | RR (bmp) | C_w_/C_L_ | WOB_vt_(RR,C_w_/C_L_) vs T_1_/T_1_ |
| 0.491 | 35 | 8.3±2.1 | 0.527 | 35 | 5 | 0.915 |
|  |  |  |  | 40 |  | 0.908 |
|  |  |  |  | 45 |  | 0.883 |
|  | 40 |  | 0.803 | 50 |  | 0.95 |
|  |  |  |  | 55 |  | 0.879 |
|  |  |  |  | 35 | 8 | 0.948 |
|  | 45 |  | 0.602 | 40 |  | 0.972 |
|  |  |  |  | 45 |  | 0.924 |
|  |  |  |  | 50 |  | 0.909 |
|  | 50 |  | 0.917 | 55 |  | 0.91 |
|  |  |  |  | 35 | 12 | 0.946 |
|  |  |  |  | 40 |  | 0.96 |
|  | 55 |  | 0.761 | 45 |  | 0.9 |
|  |  |  |  | 50 |  | 0.946 |
|  |  |  |  | 55 |  | 0.785 |
|  | Mean±SD | | 0.722±0.157^#^ | Mean±SD: | | 0.916±0.046*^,#^ |
| PIP vs T_1_/T_1_ | RR (bmp) | C_W_/C_L_ | PIP(RR) vs T_1_/T_1_ | RR (bmp) | C_W_/C_L_ | PIP(RR,C_W_/C_L_) vs T_1_/T_1_ |
| 0.388 | 35 | 8.3±2.1 | 0.449 | 35 | 5 | 0.87 |
|  |  |  |  | 40 |  | 0.911 |
|  |  |  |  | 45 |  | 0.877 |
|  | 40 |  | 0.608 | 50 |  | 0.915 |
|  |  |  |  | 55 |  | 0.954 |
|  |  |  |  | 35 | 8 | 0.958 |
|  | 45 |  | 0.506 | 40 |  | 0.947 |
|  |  |  |  | 45 |  | 0.925 |
|  |  |  |  | 50 |  | 0.872 |
|  | 50 |  | 0.8 | 55 |  | 0.923 |
|  |  |  |  | 35 | 12 | 0.947 |
|  |  |  |  | 40 |  | 0.934 |
|  | 55 |  | 0.74 | 45 |  | 0.886 |
|  |  |  |  | 50 |  | 0.933 |
|  |  |  |  | 55 |  | 0.882 |
|  | Mean±SD: | | 0.621±0.149^#^ | Mean±SD: | | 0.916±0.031*^,&^ |
| MAP vs T_1_/T_1_ | RR (bmp) | C_W_/C_L_ | MAP(RR) vs T_1_/T_1_ | RR (bmp) | C_W_/C_L_ | MAP(RR,C_W_/C_L_) vs T_1_/T_1_ |
| 0.47 | 35 | 8.3±2.1 | 0.785 | 35 | 5 | 0.895 |
|  |  |  |  | 40 |  | 0.943 |
|  |  |  |  | 45 |  | 0.858 |
|  | 40 |  | 0.688 | 50 |  | 0.9 |
|  |  |  |  | 55 |  | 0.968 |
|  |  |  |  | 35 | 8 | 0.949 |
|  | 45 |  | 0.874 | 40 |  | 0.968 |
|  |  |  |  | 45 |  | 0.908 |
|  |  |  |  | 50 |  | 0.853 |
|  | 50 |  | 0.822 | 55 |  | 0.945 |
|  |  |  |  | 35 | 12 | 0.748 |
|  |  |  |  | 40 |  | 0.639 |
|  | 55 |  | 0.793 | 45 |  | 0.641 |
|  |  |  |  | 50 |  | 0.877 |
|  |  |  |  | 55 |  | 0.641 |
|  | Mean±SD: | | 0.792±0.068* | Mean±SD: | | 0.849±0.121* |
| Z - respiratory system impedance, T_1_/T_1_ – ventilation inhomogeneity degree, RR – respiratory rate, C_W_/C_L_ – chest wall compliance to lung compliance ratio, WOB_vt_ – work of breathing, PIP – peak inspiratory pressure, MAP – mean airway pressure,*P<0.001, ^&^P<0.005, ^#^P<0.05. There were compared Z vs Z(RR), Z vs Z(RR, C_W_/C_L_) and Z(RR) vs Z(RR, C_W_/C_L_) and analogically for WOB_vt_, PIP and MAP. | | | | | | |


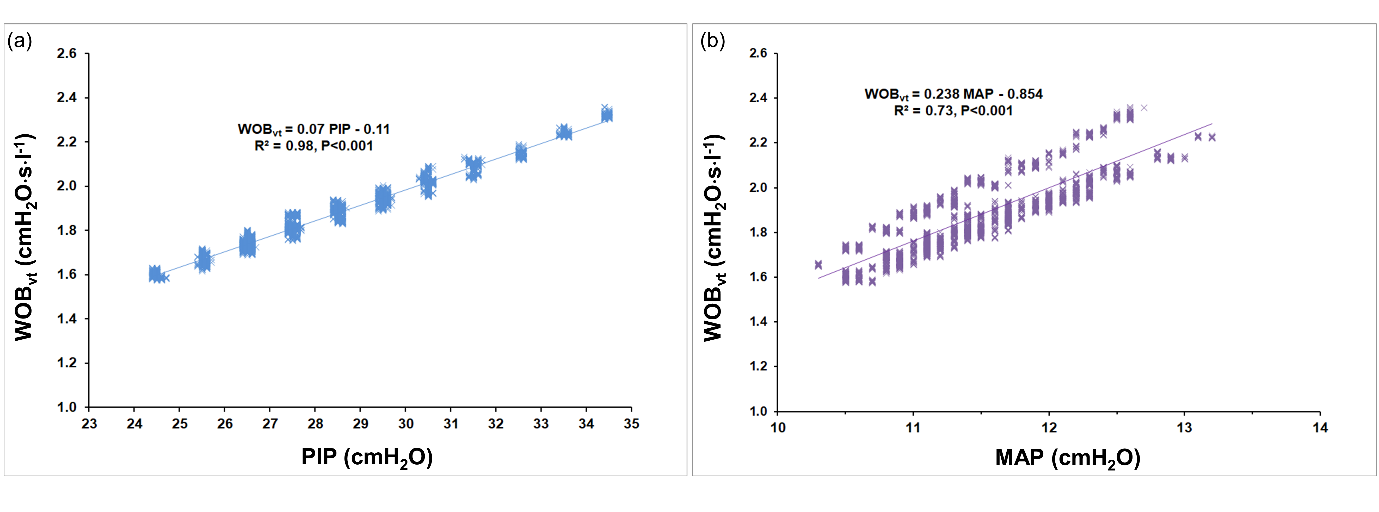


**Figure S1.** Simulation results: work of breathing (WOB_vt_) versus peak inspiratory pressure (PIP) (**a**) and mean airway pressure (MAP) (**b**) during pressure-controlled ventilation of patient P_3_ (R_rs_= 205 cmH_2_O·s·l^-1^C_rs_=1.14 ml·cmH_2_O^-1^, C_w_/C_L_: 5, 8 and 12).
